# Smartphone Sensing of Road Surface Condition and Defect Detection

**DOI:** 10.3390/s21165433

**Published:** 2021-08-12

**Authors:** Dapeng Dong, Zili Li

**Affiliations:** 1 Department of Computer Science, Maynooth University, W23 F2H6 Maynooth, Ireland; 2 Discipline of Civil, Structural and Environmental Engineering, School of Engineering, University College Cork, T12 K8AF Cork, Ireland; 3 Science Foundation Ireland, Irish Centre for Applied Geoscience (iCRAG), D04 V1W8 Dublin, Ireland

**Keywords:** road surface condition, smartphone sensing, unsupervised machine learning, defect detection

## Abstract

Road surface condition is vitally important for road safety and transportation efficiency. Conventionally, road surface monitoring relies on specialised vehicles equipped with professional devices, but such dedicated large-scale road surveying is usually costly, time-consuming, and prohibitively difficult for frequent pavement condition monitoring—for example, on an hourly or daily basis. Current advances in technologies such as smartphones, machine learning, big data, and cloud analytics have enabled the collection and analysis of a great amount of field data from numerous users (e.g., drivers) whilst driving on roads. In this regard, we envisage that a smartphone equipped with an accelerometer and GPS sensors could be used to collect road surface condition information much more frequently than specialised equipment. In this study, accelerometer data were collected at low rate from a smartphone via an Android-based application over multiple test-runs on a local road in Ireland. These data were successfully processed using power spectral density analysis, and defects were later identified using a *k*-means unsupervised machine learning algorithm, resulting in an average accuracy of 84%. Results demonstrated the potential of collecting crowdsourced data from a large population of road users for road surface defect detection on a quasi-real-time basis. This frequent reporting on a daily/hourly basis can be used to inform the relevant stakeholders for timely road maintenance, aiming to ensure the road’s serviceability at a lower inspection and maintenance cost.

## 1. Introduction

In many countries around the world, ageing road pavement deterioration has been widely observed across vast transportation networks of both highways and rural roads. This long-term deterioration affects not only the safety and satisfaction of the road users, but also the economic efficiency. In Ireland, for example, over 99% of all goods were transported by road in 2016, leaving less than 1% to be transported by rail. As the transportation of goods plays a vital role in the Irish economy, the government invested 55% of its land transportation budget of EUR 1.9 billion to the repair and maintenance of the Irish road system in 2018 [1]. The Irish government has increasingly invested more in road improvement and maintenance over the past decade. In particular, the non-national roads covering regional and rural areas account for 94% of the entire Irish road network [2]. This lifeline road infrastructure deteriorates with time, subject to various natural and artificial causes (e.g., flooding, landslide, or new construction), which necessitates timely monitoring and maintenance to mitigate any risk.

To monitor the conditions of road surfaces, a common practice for the road asset owners is to conduct multiannual surveys using specialised equipment (e.g., ground-penetrating radar (GPR) and laser systems). Nevertheless, it is usually costly to employ these vehicles and the personnel to operate and maintain the specialised equipment. In practice, the budget for large-scale road inspection and maintenance can be limited and, therefore, necessitates novel road monitoring and repair optimisation methods [3]. Such a challenge urges more cost-effective solutions for road surface condition monitoring and defect detection on a more frequent basis.

In theory, most road surface defects may cause vehicles to vibrate, and induced vibration may technically be detectable by an accelerometer. Most smartphones manufactured today are embedded with accelerometers and other hardware sensors, which enables smartphones to collect large amounts of data at a much lower cost and higher frequency than specialised equipment. As such, the smartphone technology can also be supplementary to regular road inspection, i.e., it can extend the regular examination period, thus reducing the overall cost in the long run [4]. Recently, some studies have attempted to gather small-scale data on road surface condition using smartphones, and then manually label part of the data against observed road defects to train a supervised machine learning algorithm [5]. Such a supervised algorithm, however, requires a large amount of manually labelled data for training, which can hardly apply to large-scale road inspection and data analysis.

In this study, an Android-powered smartphone was fixed to the dashboard of a test vehicle to detect road surface conditions and defects. The collected data were analysed using the power spectral density (PSD) analysis technique in order to remove noises generated from various sources—for example, car engines and suspension systems. Unlike the supervised machine learning adopted in previous efforts, an unsupervised machine learning algorithm—i.e., *k*-means—was adopted in this study to detect anomalies and defects along the monitored road with the corresponding GPS coordinates. The analysed road condition data can be reported to the asset owners and stakeholders on a frequent hourly/daily basis for timely maintenance at a lower inspection cost.

Economically, smartphones already available from road users were used for pavement defect monitoring, thus avoiding the extra cost of smartphone purchase. It is envisaged that some road users (e.g., bus drivers, commuter cars, road stakeholders) may enable their smartphones for road defect monitoring in light of their own interests.

The rest of this paper is organised as follows: Section 2 provides a brief review of the representative literature on road surface condition monitoring and the state-of-the-art techniques. Section 3 outlines the methodology adopted in this study for the field experiments and associated experimental parameters. Section 4 details the processing and analysis of gathered smartphone data. Section 5 evaluates the performance of the proposed smartphone sensing method against defect data points labelled manually. Lastly, Section 6 concludes the paper and gives some insights into further study.

## 2. Literature Review on Road Condition Monitoring

In road condition surveys, a variety of sensing technologies has been made available in the civil and transportation industry, mainly including the following three categories: (1) mechanical wave, (2) electromagnetic wave, and (3) image-based techniques [6].

The first category utilises specialised ultrasonic or acoustic sensors that transmit mechanical waves to measure the road profile [7,8], whilst the electromagnetic wave techniques depend on a series of professional equipment—for example, ground-penetrating radar (GPR) [9], light detection and ranging (LiDAR) [10], and laser systems [11]. In addition to wave measurements, the image-based techniques of the third category enable the detection of road markings, features, and surface defects. The images and videos of the road surface are usually recorded using high-quality digital cameras equipped in vehicles, and the collected data can then be automatically segmented and classified using machine learning algorithms (e.g., random forest algorithms [12] and convolutional neural networks).

Almost all the sensing technologies mentioned above rely on specialised vehicles equipped with specialised sensors (e.g., GPRs, high-quality cameras), which can be time-consuming and expensive. In the interest of time and cost, hundreds of miles of road are only surveyed on a multiannual basis, which can hardly monitor the deterioration over time of roads subjected to weather and seasonal change. Furthermore, road structural conditions may degrade rapidly within days or weeks due to landslide, subsidence, or other geohazards. Hence, a more frequent road condition monitoring system is desired in order to give an early warning of critical road damage and reveal the mechanisms of time-dependent road deterioration.

Unlike the aforementioned expensive monitoring methods using specialised sensors and vehicles, low-cost general sensors available to the general public (e.g., smartphones) have also attracted some attention for the detection of road surface condition. Recently, Sattar et al. [4] conducted an in-depth review of over 19 previous studies on smartphone sensing of road surface condition using different data analysis methods. In this research area, earlier studies such as [13,14] have opted for threshold-based heuristic detection methods. Mohan et al. [13] set a threshold limit called Z-Peak to detect accelerometer signals above a given value. Furthermore, Mednis et al. [14] advanced the practice by setting an additional threshold called Z-Diff, whereby they looked not only at the amplitude of the accelerometer, but also at the differences in the signal data from one data point to the next, using an algorithm called STDEV (Z).

More recent studies started to focus on developing machine learning techniques for analysing smartphone data road anomalies. In [5], Allouch et al. compared three different types of machine learning techniques in terms of their performance of smartphone data analysis. They found that the C4.5 decision tree algorithm performed remarkably better than the naive Bayes classifier and the support vector machines. In particular, results were achieved by training the data after pre-processing via a correlation-based feature selection process.

However, among all of the available machine learning techniques, there is a lack of investigation of unsupervised algorithms for road surface condition monitoring and defect detection. To date, only one research group—Bhoraskar et al. [15]—has specifically investigated the techniques of unsupervised machine learning for the detection of road defects and surface conditions. Nevertheless, Bhoraskar et al. focused on bump and vehicle braking detection for traffic conditions rather than different types of road structural defects. To this end, this study aims to integrate unsupervised machine algorithms into smartphone sensors and signal-processing techniques to offer a cost-effective solution to road surface inspection.

In most previous studies, the smartphone sensor data sampling frequency was high (e.g., Allouch et al. used 50 Hz [5]) in order to gather large amounts of road surface data. However, high sampling frequency will lead to high battery power consumption in the smartphone, which hinders the application of smartphone sensing to many road users at a larger scale. To this end, a low sampling rate of 16 Hz is adopted in this study for the envisaged wide-application scenarios suitable for the general public.

In the next step, the data gathered by multiple smartphones from many road users (e.g., bus drivers, commuter cars) can then be analysed for road surface defect detection using an offline algorithm at this early research stage. At present, alternative online approaches (e.g., Kalman filter methods) usually require smartphone data to be gathered at very high sampling rate. Such real-time smartphone sensing and data analysis will consume significant battery power, which can hardly be accepted by many road users.

## 3. Smartphone Data Acquisition

### 3.1. Project Background

In this study, a segment of national road (R148) in County Kildare, Ireland, was chosen as the test road. The main reasons for selecting R148 as the test road rather than a national road or motorway are listed as follows:

- The defects and associated GPS coordinates of the test road need to be recorded through manual visual inspection by researchers in order to validate the proposed defect detection algorithm. It is usually more challenging and riskier to conduct a visual inspection on a busy motorway (e.g., M50) than on a regional road;
- In a simplified driving scenario, the test vehicle is designed to maintain a relatively constant speed (e.g., 30 ± 5 km/h) during data collection. Such a controlled road environment is more likely to be achieved on a regional road with few road users than on a busy motorway;
- In Ireland, the non-national roads covering regional and rural areas account for 94% of the entire Irish road network [2], which deserves specific investigation for road monitoring technology;
- Following the development of smartphone sensing based on regional roads, future studies may apply this technology to many more road types in a transportation network, including national roads, motorways, bridges, tunnels, etc.

**Figure 1:**
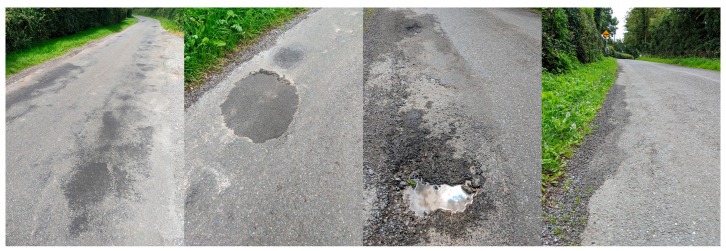
Photo images of several typical road surface defects were taken from the test road segment. From left to right, they show road surface distortion, patching, pothole, and rutting.

The test road is approximately 2.2 km in length. It contains several typical kinds of surface defect, in accordance with the Pavement Surface Condition Index (PSCI) [16]. In the PSCI manual, four categories of road surface condition on rural flexible roads are defined, including surface defect, pavement deformation, crack, and surface opening. A number of photo images were taken from the test road segment to illustrate the types of defects and deteriorations of interest to this study, as shown in Figure 1. The images show road surface distortion, patching, pothole, and rutting, from left to right. The severity of the defect can be rated from level 10 (no obvious defect) to level 1 (extensive structural distress). The rating is measured based on the size of the defect—for example, the diameter of a pothole or the length of rutting [16]. For brevity, this study focuses on the feasibility of using a smartphone to detect the existence of road surface defects at a given location marked by GPS coordinates, rather than classifying those defects into different types.

### 3.2. Data Collection

Road condition data were collected using a smartphone (Google Pixel 2, hardware version 1) running an Android operating system (version 10). The smartphone was fixed on the dashboard of the test vehicle so as to eliminate the need for complex orientation calibration algorithms (one potential solution was mentioned in [13]). During data collection, the test vehicle was maintained at a relatively constant speed of 30 ± 5 km/h. The test road was traversed eight times in each direction, and data were collected independently from each run. As the test road is a two-way road, data collected in each direction were used for independent analysis.

Of all the gathered smartphone data, the two most important datasets for road condition analysis were the accelerometer readings and the GPS coordinates. The accelerometer reading data, including the x-, y- and z-axis readings, were directly exported from the smartphone hardware sensor (STMicroelectronics LSM6DSM sensor) of the Android SensorManager. In the field experiments, the z-axis readings were used for analysis, as they determine most of the vertical vibrations of the vehicle when driving on an uneven surface. The accelerometer data were sampled at a constant rate of 16 Hz, which is much lower than that adopted by previous investigations—for example, Allouch et al. used 50 Hz [5], Mohan et al. used 310 Hz [13], and Eriksson et al. used 380 Hz (specialised accelerometer) [17].

The adoption of a low sampling rate was mainly attributed to the envisaged application scenarios. In this study, the application was designed to be used by the general public, i.e., people from the residential communities of the interested areas would be encouraged to use the developed application on a daily basis, with acceptable battery power consumption at a low sampling rate of 16 Hz. Due to the low sampling rate (approximately one sample per 0.5 m), a relatively small defect could easily be missed by one or two runs. To this end, the collected data could be collectively cross-validated with repetitive runs from multiple users—for example, community commuters and buses travelling on the same segments of road every day. Due to the limited demand for a 100% real-time monitoring system for road surface condition, such gathered big data from multiple users can then be processed using batch analysis on a daily/hourly basis. On the other hand, another undeniable reason for the much lower sampling rate is battery power conservation. With a much higher sample rate configured, batteries could be drained very quickly by data acquisition and transmission. Moreover, the larger amount of data generated by a higher sampling rate is more likely to be restricted by smartphones’ local storage capacity.

Given a constant driving speed *S_d_* and a fixed sampling rate *r_s_*, an accelerometer reading was taken for every $\frac{S_{d}}{r_{s}}$ meters. This configuration in the experiments allowed an accelerometer reading to be taken for every ~0.5 m on average. Given the 2.2-km-long test road, there were ~4500 data points collected per test run. In total, there were approximately 36,000 data points of vertical vibration and GPS collected in this study.

The GPS coordinates for the defects were first recorded through manual visual inspection along the test road. The assumed accuracy of the GPS readings was based on the official GPS report (https://www.gps.gov/systems/gps/performance/accuracy/ (accessed on the 1 June 2021)), i.e., typically a ~4.9-m radius under an open-sky environment [18]. Thus, defects were roughly labelled with ±4.9-m distance from the central point of recording. The manually labelled defects and their corresponding GPS readings were used as the baseline for assessing the accuracy of the developed smartphone sensing method. In the smartphone sensing experiments, GPS coordinates were collected along with the accelerometer readings.

## 4. Data Analysis for Road Defect Detection

### 4.1. Data Processing

**Figure 2:**
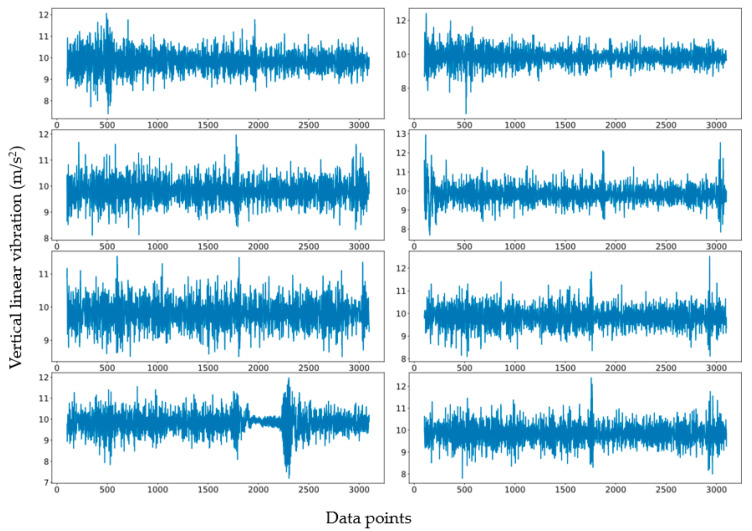
The collected accelerometer raw data (vertical linear vibration in the vertical axis and data points in the horizontal axis) from eight repeated and independent runs in one direction.

Figure 2 shows collected accelerometer readings along the road distance from eight repeated and independent runs in one direction. Inevitably, the data are noisy, and the number of data points varies slightly due to the inconsistent starting/stopping positions of the test vehicle in each test run.

The aim here was to extract the abnormal events that correspond to various road surface defects. When these defects were recorded by the accelerometer sensor—i.e., the vertical vibrations detected—the corresponding sensor readings were considered as the events of interest. On the other hand, any periodic signals that are likely to be introduced by a vehicle’s engine vibration and/or suspension systems were of no interest to the study; hence, the noise. In the worst case, these periodical signals can easily bury those small vibrations caused by minor defects in the background, rendering them very difficult to observe. This issue becomes particularly critical when the smartphone is fixed on the test vehicle and, as such, the vibrations generated by the car components (e.g., engine, suspension system, dashboard, etc.) are multiplexed.

**Figure 3:**
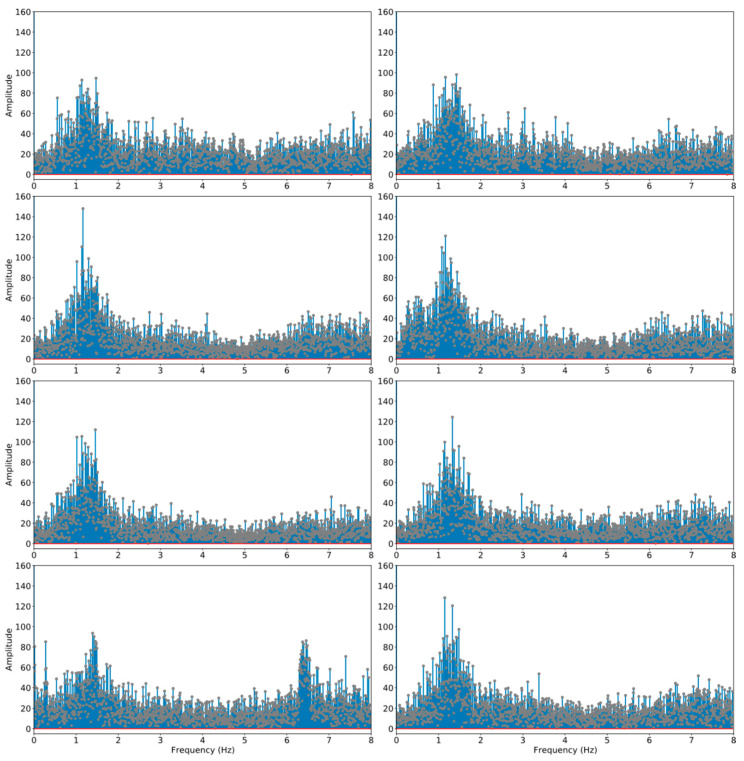
The PSD analysis of the eight independent test runs in one direction. The results from the PSD analysis revealed the predominant frequencies from the raw data. The stem plots show that the predominant frequencies are clustered around the frequency band (1–2) Hz, which are the typical frequencies generated by most cars’ suspension systems. Additionally, there are smaller clusters and relatively flat predominant frequencies across the x-axis; this is due mainly to the vibrations generated by the car engines and dashboards.

To understand how these periodical signals present in the collected data, a power spectral density (PSD) analysis was conducted. Figure 3 shows the stem plot of the temporal signals transformed into frequency domain. PSD analysis was applied to each independent test run; this was done by first computing a fast Fourier transform (FFT) of the signal ($\hat{f}$) then multiplying $\hat{f}$ by its conjugate to receive the magnitude of $\hat{f}$ squared—i.e., ${\left|\hat{f}\right|}^{2}$—which is the vector of the power of each frequency; then, it was normalised with frequency bin width. The analysis results show that the collected raw data contain some predominant frequencies that were likely to be periodical. In particular, there is a cluster of frequencies grouped around band (1–2) Hz; this perfectly matches the typical natural frequencies of most cars’ suspension systems [19]. In addition, there are several smaller clusters of dominant frequencies throughout the frequency band; these are likely due to the engine and dashboard vibrations, as these car components are common sources for noises in the frequency band (1–9) Hz [20]. These patterns can be noted repetitively in each independent test run. In order to de-noise the raw data, these periodical frequencies introduced by the test vehicle need to be removed. Moreover, it should be noted that the extraordinarily high amplitude of some frequencies is very likely due to harmonics.

**Figure 4:**
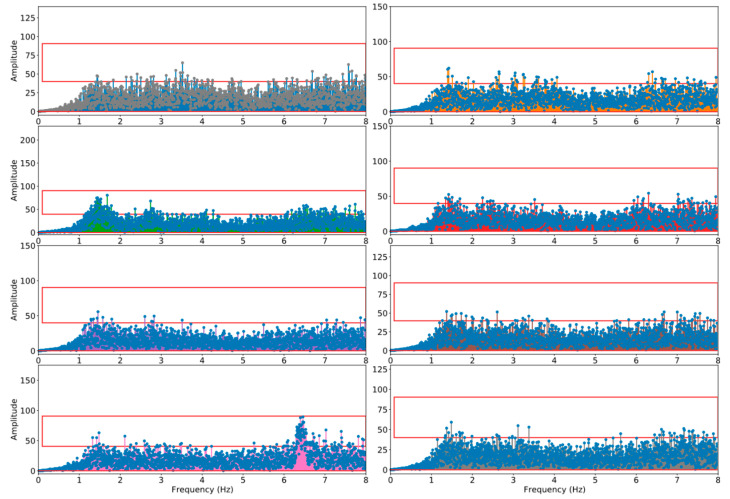
Filtered signals after the removal of periodical signals.

To remove these unexpected frequencies, a threshold (τ) needs to be determined first. Referring to the previous research results in [19,20], and based on the empirical studies conducted in this work, a threshold was settled at the average amplitude of the signals. In fact, the actual threshold may vary slightly depending on the vehicle types, the vehicle models, and the placement of the smartphones. A dedicated threshold (τ) can be adjusted according to the relative value of the amplitude of each individual datum collected. Once the τ is determined, those frequencies can be removed by simply zeroing out all the frequency powers that are greater than tau, as shown in Figure 4. By applying an inverse FFT to the filtered signals, a filtered signal can therefore be reconstructed.

Furthermore, considering that car engine noises oscillate, in general, at higher frequencies, as well as other types of noise generated by mechanical components during driving, the high-frequency signals should also be filtered. Nevertheless, as the sample rate is significantly lower compared with other aforementioned research, all signals are distributed in the frequency band (1–9) Hz, overlapping with noise signals (e.g., from car engines and suspension systems).

It would be very challenging to extract information of interest from the mixed signals without a carefully and artificially crafted signal-processing mechanism. Without compromising generality, when the test vehicle passed over a defect, an abnormal signal should be trigged by both the front wheel and the rear wheel. The distance between a vehicle’s front axle and the rear axle is often known as the wheelbase. A typical wheelbase for cars ranges from 1.2 to 3 m. Given a driving speed of *S_d_* and the wheelbase *B*, it is expected to observe a mirrored signal in a $\frac{B}{S_{d}}\;$delay. These paired signals are the primary subjects of interest in the experiments. Given the driving speed of 30 ± 5 Km/h and a standard wheelbase, the paired signals should appear within frequency band (0.1–0.5) Hz.

The intention is to keep the frequency band narrow enough so that the filtered signals only exhibit the characteristics of defect-related events. The boundary frequencies are also known as the cutoff frequencies. A first-order Butterworth filter [21] was chosen experimentally for the filtering process, i.e., a bandpass for frequency band (0.1–0.5) Hz.

**Figure 5:**
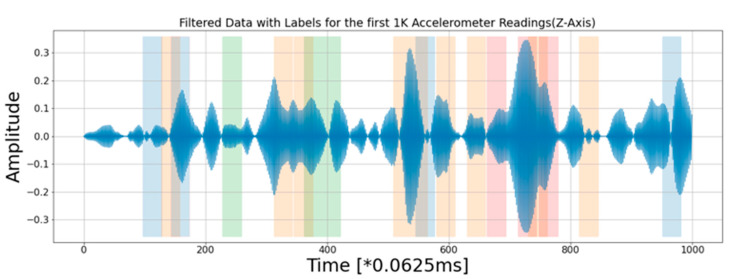
Filtered signal with labels.

**Figure 6:**
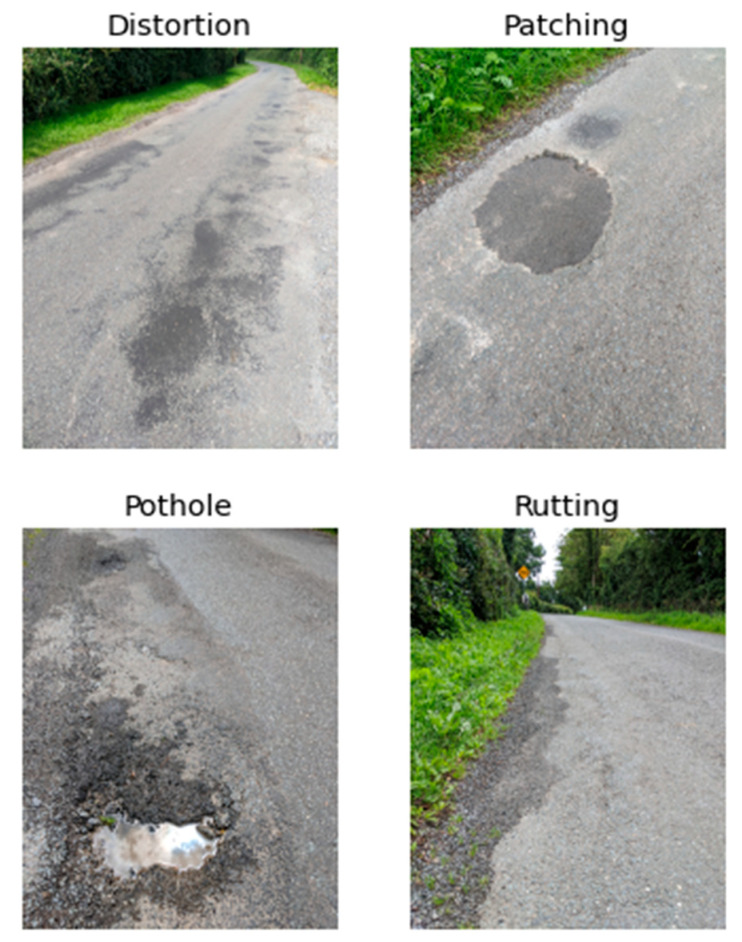
Examples of defects observed from the designated road segment.

With the average driving speed of 30 ± 5 Km/h and a 16-Hz sampling rate, Figure 5 shows the road surface condition of the first 500 m, and the corresponding accelerometer readings. The vertical bars in various colours indicate the manually labelled defects, including potholes (light blue), distortion (light orange), rutting (light green), and patching (pink). It is now much easier to observe how the accelerometer readings reflect the abnormal events corresponding to many of the actual defects. The width of the vertical bars indicates an approximate region where the defect was observed; this was done by mapping GPS readings in the collected data to the GPS coordinates recorded in the photos taken at the site of the defects. However, due to the level of accuracy of GPS for civilian use, the GPS coordinates recorded by different devices and at a different travelling speed may not be an exact match. Thus, in order to accommodate the potential drifts at a certain level, a defect is marked in a range. In this work, the width of the vertical bar corresponds to 4.9 m (or higher, depending on the environment); this is the main reason that the vertical bars shown in Figure 5 have some overlaps. An example of each type of defect is shown in Figure 6.

To summarise the analysis processes, the data pre-processing stage consists of three essential steps.

1. PSD analysis for the identification of periodical signals introduced by the test vehicles;
2. Filtering out periodical signals and low frequencies;
3. Reconstructing signals using inverse FFT.

### 4.2. Defect Detection

Autonomous defect detection without the involvement of human inspection relies on emerging machine learning algorithms. Broadly speaking, a machine learning algorithm can be classified as either supervised or unsupervised. A supervised machine learning algorithm generally requires a significant amount of labelled data for training. The labelled data must present various types and characteristics of defect, so that the trained algorithms can be generalised to recognise similar defects. In contrast, an unsupervised machine learning algorithm does not require pre-knowledge about defects. Based on the characteristics of the defects contained in the dataset, the algorithm can automatically classify them into a predefined number of categories. In the application scenario of this work, collection and manual labelling of defects can be very time consuming and costly. An unsupervised machine learning *k*-means algorithm was adopted, as this is one of the most well-developed and widely accessible clustering algorithms. Future study will evaluate the performance of different clustering algorithms for road defect classification.

Assuming that the collected accelerometer readings can be organised into a set of observations (x_1_, x_2_, …, x_n_), where each observation is a d-dimensional feature vector, the aim of the use of a *k*-means algorithm in this work was to classify the n observations into k sets, S = {s_1_, s_2_, …, s_k_}, (k ≤ n). The objective of a *k*-means algorithm, in general, is to minimise the within-set Euclidean distance between x and the s_j_ centroid µ_j_.

**Figure 7:**
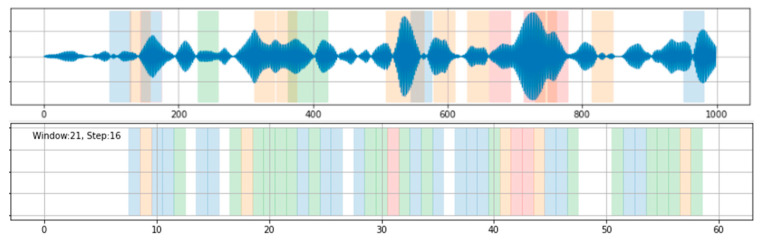
Classification results from *k*-means with various window sizes and step sizes for k = 5.

The first step for employing a *k*-means algorithm in this work was to identify what constitute “features”. A scalar data point is obviously lack of identity for characterising defects. An intuitive choice is to use a number of consecutive data points that span a short timeframe, i.e., a window of data points. In the second step, the number of categories needs to be defined. In practice, there were four types of defects—i.e., pothole, distortion, rutting, and patching—commonly observed along the road in this study. Depending on the severity level of the defect, potholes are mostly represented by sharp spikes with different levels of amplitude. In contrast, rutting, distortions, and patching are all reflected as long-spanning blocks. The situation could be further complicated when multiple different defects were in close proximity. Figure 7 shows the classification results with various window sizes ~21 (10 m) and step size of 16 (1-s readings, 7.7 m) for k = 5; as can be seen, there are many misclassifications. This is also evidence that a window of data points cannot adequately characterise so many types of defects, with such a variety of shapes, lengths, depths, diameters, etc. However, the situation may be improved by increasing the density of the data points—i.e., increasing the sampling rate for data collection—but considering the battery life of smartphones, their available storage space, and the time required to upload data for analysis, a sampling rate in the <32-Hz range is more practical.

A compromised solution is to only detect defects at a given location and classify them into one of two types: narrow spikes, or long-spanning blocks. In such a configuration, only three categories (narrow spikes, long-spanning blocks, and without a defect) would suffice and, as such, k is set to be 3.

**Figure 8:**
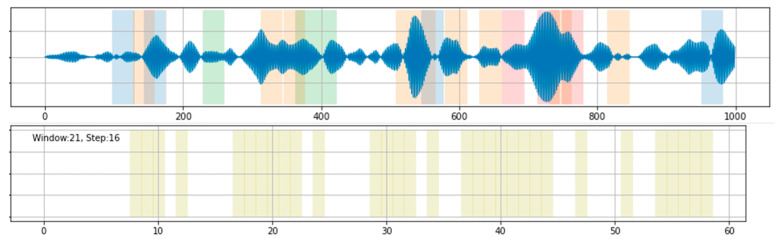
Classification results from *k*-means with various window sizes of ~10 m and step sizes of 1 s for k = 3.

Furthermore, the feature vector for each sliding window includes the robust measure of central location (i.e., the median) and the dispersion of data points in the sliding window (i.e., the 310 variance). As shown in Figure 8, the classification results have largely improved. Of particular interest is that the signal (Figure 8, upper) may contain some irrelevant events, possibly caused by unusual driving manoeuvers. In order to minimise such events, a cross check between independently collected data is needed.

## 5. Evaluation of the Proposed Method

In this section, the performance of the proposed scheme is evaluated against 16 independently collected data points. The measure of accuracy and precision is defined. The performance measures are computed from the confusion matrix, in terms of accuracy, recall, precision, and f-measure. Thus, it is important to clarify how the terms true positive (TP), false negative (FN), true negative (TN), and false positive (FP) are defined in this application scenario.

**Figure 9:**
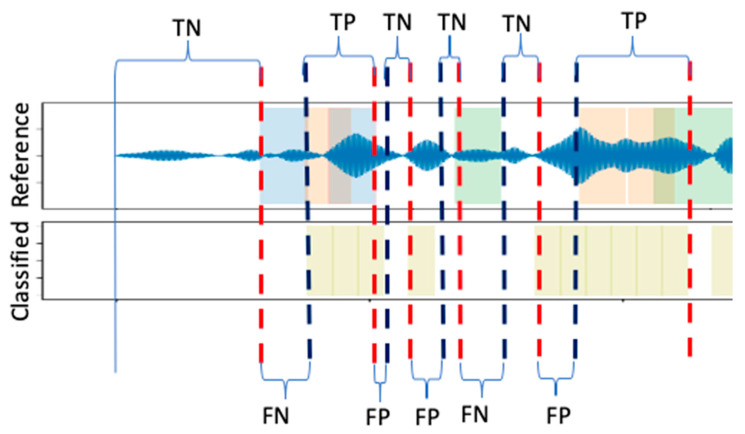
Illustration of the evaluation scheme.

Referring to Figure 8, the manually labelled defect blocks are treated as references. Each reference may contain one or more continuous labelled blocks. For example, the first reference block contains three labelled blocks, starting from GPS coordinate (Latitude: 53.4060605, Longitude: −6.5243956) to (Latitude: 53.4063506, Longitude: −6.524446), within approximately 37 m. There are a total of 30 reference blocks with various lengths. Starting from the first cross-validated classification result, each classified block is checked as to whether it falls into one of the reference blocks. If it partially overlaps with one of the reference blocks, then the length of the overlapping part and the length of the non-overlapping part will be recorded. Accumulating these measures, the TP measure is the length of the correctly classified road segments that contain defects; the TN measure is the length of the correctly classified road segments that do not actually contain defects; the FP measure is the length of the road segments incorrectly classified as containing defects, but actually containing no defects; whilst the FN measure is the length of the road segments incorrectly classified as not containing defects, but containing defects. The scheme is illustrated in Figure 9.

**Figure 10:**
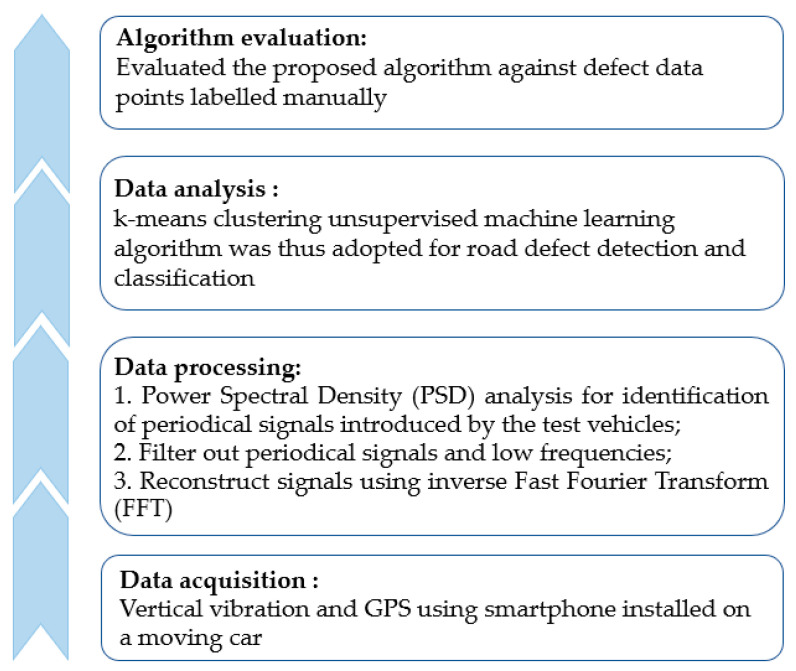
The outline of the proposed smartphone sensing of road surface condition.

Furthermore, to minimise the effects of GPS drift, 16 sets of data points were independently collected. Classifications were also done for each dataset, independently. For each classification, the result (on a per dataset basis) was cross-validated with two other randomly selected classification results. In summary, the outline of this study can be found in Figure 10, while Table 1 shows the accuracy, recall, precision, and *f*-measure for each run of the evaluation.

Our results show that the proposed method achieved a maximum accuracy of 87% and an average accuracy of 84%. More importantly, the recall that indicates the ratio of the total number of correctly classified positive (defects) examples divided by the total number of actual defects reached 98%, and was 87% on average. The high recall indicates that more defects have been correctly classified, with a relatively small number of false negative results. Nevertheless, the precision is relatively low—52% at maximum and 46% on average. This indicates that the proposed method classified a relatively high number of false positives. The high recall and low precision indicate that the proposed method was able to correctly identify most of the defects, but erroneously classified a number of normal road segments as containing defects. In the last column of Table 1, the *f*-measure shows the relative value of precision and recall by using harmonic mean instead of arithmetic mean to diminish the weight of extreme values. In addition, the confusion matrix for the evaluation is shown in Table 2. Since multiple independent evaluations were conducted, the values shown in the table are the average values across the evaluations shown in Table 1.

In addition, an ROC curve (receiver operating characteristic curve) may be considered in order to validate the proposed approach in this study [22]. In general, it is straightforward to construct an ROC curve for a classifier with univariate output. However, the *k*-means algorithm adopted in this study does not transform the multivariate feature input into a univariate output; as such, it will require dedicated investigation to construct an ROC curve for the *k*-means algorithm in future works, as stated in [23].

Previous investigations into the smartphone sensing of road surface condition usually adopted high frequencies (e.g., 50 Hz in [5]), but were only able to detect large defects (e.g., potholes) [17]. In this study, the accelerometer sampling rate was set to a lower level at 16 Hz, which enabled it to detect not only potholes, but also other types of relatively minor defects.

## 6. Conclusions

This paper conducted a series of field experiments to collect over 36,000 data points of vertical vibration and GPS using a smartphone installed on a moving car. The gathered smartphone data were then processed and analysed to detect road surface defects. The developed smartphone sensing technique can potentially enable quasi-real-time road surface condition monitoring, improving the safety and efficiency of transportation while simultaneously reducing inspection costs.

One major challenge is to extract signals corresponding to various defects from noisy sensor data. Throughout the study, the three aspects of vital importance were: (1) data quality, (2) minimising the possible sources of background noise in the collected data, and (3) the choice of algorithms. In this study, multiple sets of data were collected independently and carefully labelled; signal data were analysed using PSD and filtered using Butterworth filters for de-noising; a carefully crafted *k*-means algorithm was used for the identification of defects.

When evaluated against manually labelled defects, the proposed method achieved a high recall, at 87%, but low precision, at 46%; that is, the proposed method was able to correctly identify most of the defects, but erroneously classified a number of normal road segments as containing defects, generally demonstrating the feasibility of smartphone sensing for detecting road surface condition.

Unlike previous relevant studies, the experiments in this study adopted a lower sampling frequency of smartphone data collection, which enabled the detection of both major and minor road defects, and at a lower rate of battery consumption.

Nevertheless, the experiments in this study were conducted in relatively idealised conditions by driving at a constant speed and fixing the smartphone at one position. Future studies will conduct many more experiments on the smartphone sensing of road surface condition, and collect a large amount of data subject to different driving speeds, behaviours, car models, smartphone types, placement of smartphones, sampling rates, and other influences.

In practice, acceleration and GPS data can be gathered by different smartphones with a variety of sensor specifications, inertia vibration, battery consumption, etc. Further investigation will evaluate and compare the data gathered by smartphones of different brands and models. Future studies will also compare smartphones using iOS and Android operating systems for accelerometer and GPS data acquisition, although in theory the difference is likely to be negligible.

In this study, only the road directly above ground is considered, but not road segments within a bridge, tunnel, or other infrastructure possibly subject to dynamic loads. For such road segments embedded in a dynamic system, eigenvalue perturbation and other online data analysis methods may be adopted for real-time damage detection. For further study, the smartphone vibration data can be combined with video/image camera data and other inspection methods to jointly monitor and assess large-scale road structural conditions with improved accuracy.

## Figures and Tables

**Table 1:** Evaluation metrics for the proposed smartphone sensing.

Evaluation	Accuracy	Recall	Precision	*f*-Measure
1	0.86	0.89	0.43	0.58
2	0.87	0.83	0.44	0.58
3	0.84	0.82	0.44	0.58
4	0.84	0.88	0.5	0.64
5	0.81	0.98	0.47	0.64
6	0.8	0.91	0.45	0.6
7	0.84	0.92	0.46	0.61
8	0.82	0.94	0.41	0.57
9	0.84	0.92	0.46	0.62
10	0.8	0.89	0.45	0.6
11	0.83	0.89	0.49	0.63
12	0.81	0.85	0.48	0.61
13	0.88	0.77	0.48	0.59
14	0.83	0.83	0.43	0.56
15	0.87	0.83	0.52	0.64
16	0.86	0.84	0.49	0.62

**Table 2:** The confusion matrix of the evaluation results. As multiple independent evaluations were conducted, the values shown in the table are the average values.

	True	False
True	32.44	37.69
False	4.63	189.25

## Data Availability

The main reported data can be found in the tables and figures in this paper.
